# Potential role of microRNA-126 in the diagnosis of cancers: A systematic review and meta-analysis

**DOI:** 10.1097/MD.0000000000004644

**Published:** 2016-09-02

**Authors:** Jin Yan, Shijie Ma, Yifeng Zhang, Chengqiang Yin, Xiaoying Zhou, Guoxin Zhang

**Affiliations:** Department of Gastroenterology, The First Affiliated Hospital with Nanjing Medical University, Nanjing, Jiangsu 210029, P. R. China (JY, YZ, XZ, GZ); The First Clinical Medical College, Nanjing Medical University, Nanjing, Jiangsu 210029, P. R. China (JY, YZ, XZ, GZ); Department of Gastroenterology, Huai’an First People's Hospital, Nanjing Medical University, Huai’an, Jiangsu 223300, P. R. China (SM); Department of Gastroenterology, Sir Run Run Hospital, Nanjing, Jiangsu 210000, P. R. China (CY).

**Keywords:** cancer, diagnosis, meta-analysis, miR-126, systematic review

## Abstract

**Background:**

Cancer has become a major public concern all over the world and early diagnosis of cancer is of great benefit for treatment and prognosis. Several studies have investigated the association between abnormal circulating microRNA-126 (miR-126) expression and the risk of various cancers, but the results are inconsistent. Therefore, this meta-analysis was carried out to assess the potential diagnostic value of miR-126 for cancer.

**Methods:**

Relevant studies were searched from PubMed, Embase, and Web of Science and we calculated the pooled sensitivity, specificity, positive likelihood ratio (PLR), negative likelihood ratio (NLR), diagnostic odds ratio (DOR), and area under the summary receiver operator characteristic curve (AUC) to assess the diagnostic value of miR-126 for cancer detection.

**Results:**

A total of 745 cancer patients and 749 controls from 11 studies of 7 papers were included in this meta-analysis. The summary estimates revealed that the pooled sensitivity was 68% (95% confidence interval [CI]: 60–75%), the specificity was 76% (95% CI: 65–85%), the PLR was 2.87 (95% CI: 1.96–4.21), the NLR was 0.42 (95% CI: 0.35–0.52), the DOR was 7 (95% CI: 4–11), and the AUC was 0.77 (95%CI: 0.73–0.80). Moreover, the sample type, cancer type, sample size, and quality score might be sources of heterogeneity.

**Conclusion:**

This systematic review and meta-analysis suggests that miR-126 has great potential to be a noninvasive biomarker in the diagnosis of cancer. However, more well-designed studies with larger sample size on the diagnostic value of miR-126 for cancer are needed in the future.

## Abstract 2

Supplemental Digital Content is available in the text

## 1 Introduction

Cancer has been a major public health problem all over the world and is considered as leading cause of mortality.^[1,2]^ Early detection of cancer is crucial for it is beneficial for both treatment and prognosis. Nowadays, histological evaluation of biopsy is the gold diagnosis standard for cancer. However, it cannot diagnose cancer at early stage and its usage is restricted in clinic for the invasive procedure.^[3]^ In the present, several serum cancer biomarkers have been used in clinic, such as carcinoembryonic antigen (CEA), carbohydrate antigens 125 (CA 125), α-fetoprotein (AFP), and so on.^[4–7]^ Nevertheless, these markers are minimally useful for early cancer screening for their relatively low sensitivity and specificity. Therefore, it is urgent to find an effective noninvasive biomarker for early detection of cancer.

Recently, numerous studies have proposed miRNAs as potential biomarkers for cancers detection. MicroRNAs (miRNAs) are noncoding single-stranded RNAs with average of 22 nucleotides and participate in many physiological processes. They bind to specific mRNA targets via base pairing at the 3′-UTR, leading to translational repression or degradation of these mRNAs.^[8]^ A wide range of cancers are reported to display significantly differential expression profiles of miRNAs compared to normal tissues and the different expression is also discovered in serum, plasma, and other body fluid.^[9–12]^ Moreover, circulating miRNAs are reported to remain stable after incubation for up to 24 hours at room temperature or after up to 8 cycles of freezing and thawing.^[13]^ Thus, miRNAs are suitable for being cancer biomarkers with stability and easily testable length.^[14]^

Among those cancer-associated miRNAs, miR-126 was first identified as an miRNA regulating human megakaryocytopoiesis.^[15]^ Further studies reported that miR-126 was involved in progression of angiogenesis, proliferation, migration, invasion, and cell survival by targeting several important genes such as CADM1, PAK4, and SOX2.^[16–18]^ So far, circulating miR-126 has been reported that it could act as a significant biomarker in the prognosis of various cancers.^[19]^ Additionally, plenty of studies have focused on the diagnosis use of circulating miR-126. Circulating miR-126 was identified as a cancer suppressor in lung cancer, malignant mesothelioma, and so on.^[20,21]^ However, other study indicated that miR-126 in plasma functioned as an oncogene in hepatocellular carcinoma and its expression was upregulated.^[22]^

Nowadays, there was still not a comprehensive conclusion for the diagnostic value of miR-126 in detecting cancers, because of different ethnicity, different cancer types, and small sample size. Therefore, the aim of our study was to conduct a meta-analysis to determine whether detection of miR-126 expression can be an effective biomarker for cancers.

## 2 Materials and methods

### 2.1 Data sources and search strategy

The meta-analysis was carried out according to the guidelines of the Preferred Reporting Items for Systematic Reviews and Meta-Analysis (PRISMA) statement and methods.^[23]^ Ethical statement is not necessary because this is a systematic review and meta-analysis which focused on the basis of published articles. The following electronic databases: PubMed, Embase, and Web of Science were searched for eligible literature until January 2016. The key words used in the research were “serum or plasma or blood or circulating” and “microRNA-126 or miRNA-126 or miR-126” and “ROC curve or diagnosis or sensitivity or specificity.” In addition, we manually searched the reference lists of eligible studies identified from the databases as well.

### 2.2 Inclusion and exclusion criteria

Two reviewers (JY and SJM) checked the abstract of the studies and read the full text if necessary to identify the final included studies. When disagreement was appeared, we discussed and consulted with the third reviewer (YFZ). Moreover, we turned to the original authors for data if necessary. Eligible studies should strictly meet the following criteria: (1) the studies utilized a case-control design and contained sufficient published data to construct 2-by-2 tables and calculate the diagnostic accuracy; (2) they detected miR-375 expression in serum or plasma; (3) cancer diagnosis was based on histopathological confirmation and healthy people or patients with benign disease were served as the control group; (4) they were published in English. In addition, articles were excluded if they were: (1) review articles, meta-analysis, letters, commentaries, and abstracts presented in conferences; (2) duplicates or continued work of previous publications; (3) studies without complete data; (4) not in English.

### 2.3 Data extraction and quality assessment

Data were retrieved from each study independently by 2 reviewers (JY and SJM) including the following characteristics: the first author, the year of publication, the country and ethnicity, sample type, cancer type, miR-375 change tendency, number of patients, diagnostic parameters, and other substantial information. Disagreements were solved by fully discussing with the third investigator (YFZ).

To ensure the quality of the meta-analysis, the Quality Assessment of Diagnostic Accuracy Studies (QUADAS) was referred to and each items in QUADAS were assessed in all the included studies.^[24]^ Two authors (JY and SJM) reached a consensus on study quality assessment, and any disagreements were resolved through a discussion with the third author (Y.F.Z).

### 2.4 Statistical analysis

All accuracy data from each study (true positives, false positives, true negatives, and false negatives) were extracted to obtain pooled sensitivity, specificity, positive likelihood ratio (PLR), negative likelihood ratio (NLR), diagnostic score, DOR, and their 95% CI. The value of a DOR ranges from 0 to infinity, with higher values indicating better discriminatory test performance.^[25]^ The SROC curve and AUC were also gathered to evaluate the diagnosis accuracy of miR-126 in cancer. An AUC value close to 1.0 implies that the test has perfect discrimination, and an AUC value close to 0.5 suggests poor discrimination.

Heterogeneity could be caused by the threshold effect, which was quantified by Spearman correlation analysis. Moreover, the nonthreshold effect was assessed by the Cochran-Q method and the test of inconsistency index (*I*^2^). A *P* value <0.05 and *I*^2^ value >50% suggest the presence of heterogeneity by the nonthreshold effect. If the non-threshold effect existed, meta-regression would be used to find out the sources. Finally, evidence of publication bias was analyzed by Deeks’ funnel plot (*P* value <0.05 was considered a significant publication bias).^[26]^ Statistical analysis was conducted utilizing Stata 12.0 (Stata Corporation, College Station, TX) and Meta-disc 1.4 (XI Cochrane Colloquium, Barcelona, Spain) software.

## 3 Results

### 3.1 Data selection

**Figure 1:**
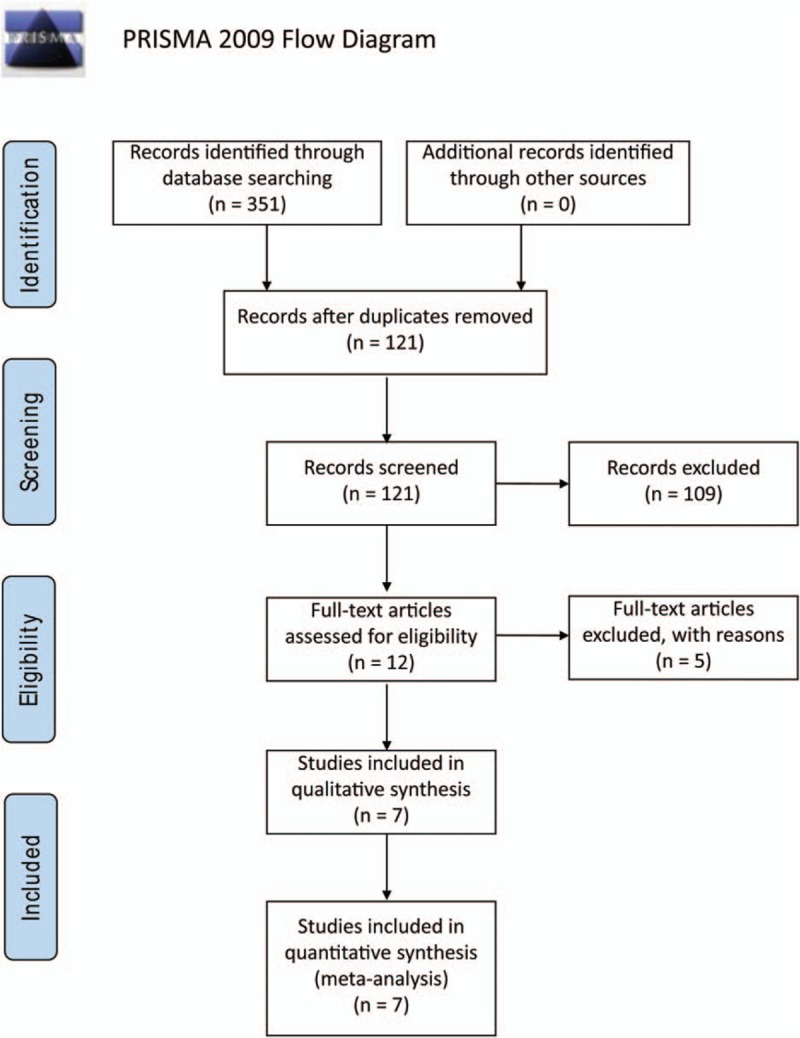
Flowchart of study selection based on the inclusion and exclusion criteria.

The initial search returned a total of 351 studies among which 120 duplicated hit. We then screened titles and abstracts, and excluded 13 reviews. Moreover, 1 study was not in English, 3 were not human associated, 56 were not related to cancer, 26 were not about diagnosis effect, and 10 were not detected in serum or plasma. Of these remained 12 literatures, their full-text versions were retrieved and 5 of them were excluded due to lack of sufficient data and rational study design. Thus, 6 high-quality literatures from independent research group met the eligibility criteria for this meta-analysis (Fig. 1).^[20–22,27–30]^

### 3.2 Study characteristics and quality assessment

All of these eligible literatures were published from 2011 to 2016 accumulating 745 cancer patients and 729 controls. The cancer patients had been histopathologically confirmed, which is gold standard for cancer diagnosis. The control individuals were from healthy volunteers who had never been diagnosed with a malignant tumor or patients with benign diseases. The study characteristics, including the first author, publication year, country, ethnicity, sample type, cancer type, miR-126 change tendency, number of patients, and diagnostic parameters, were listed in Table 1. In addition, the 7 studies were scored by QUADAS by 2 independent reviewers. Six of the 7 studies had QUADAS scores >10, indicating the reliable foundation of our analysis (Table S1).

**Table 1:**
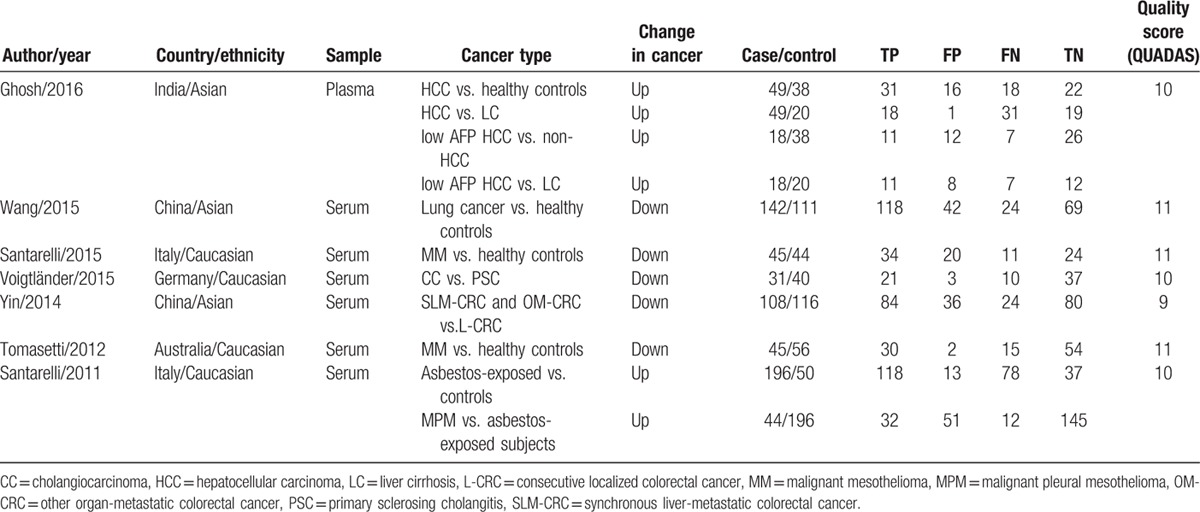
Characteristics of 7 articles included in our study that reported on using miR-126 as diagnostic biomarkers of various cancers.

### 3.3 Data analysis

**Figure 2:**
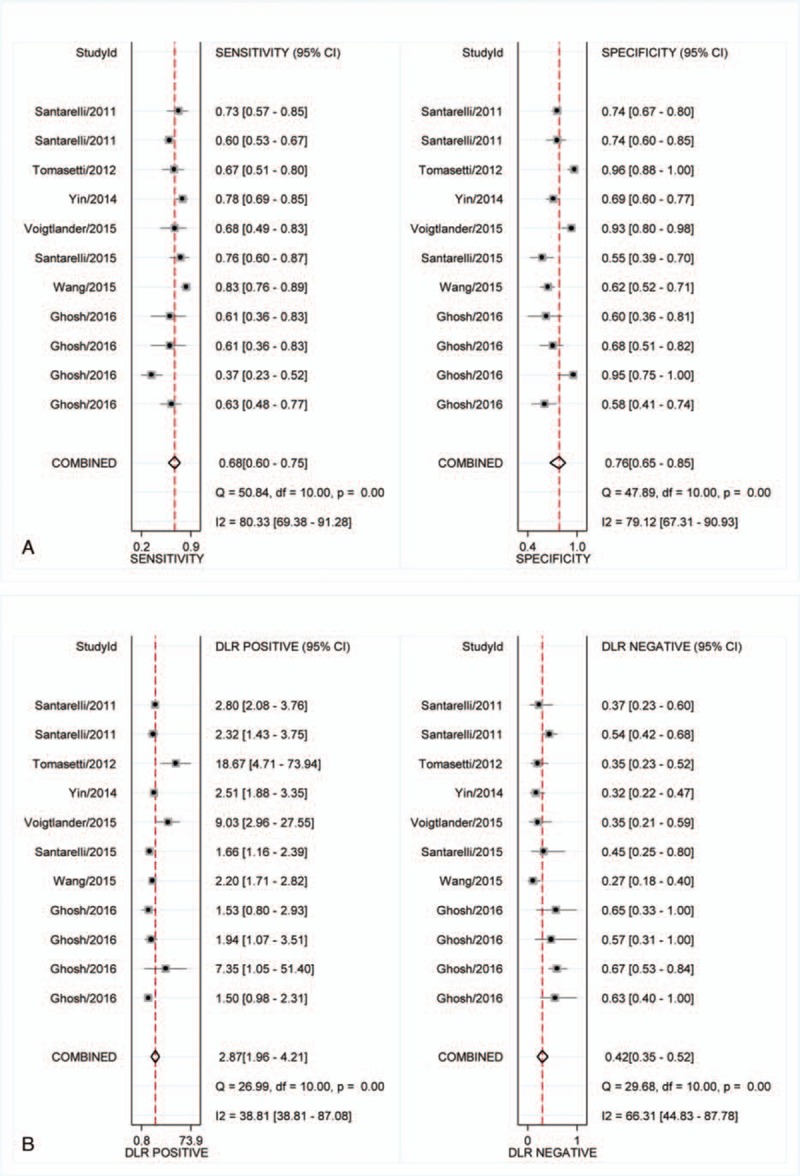
Forest plots of sensitivities, specificities, PLR, and NLR from test accuracy studies of miR-126 in the diagnosis of cancer.

**Figure 3:**
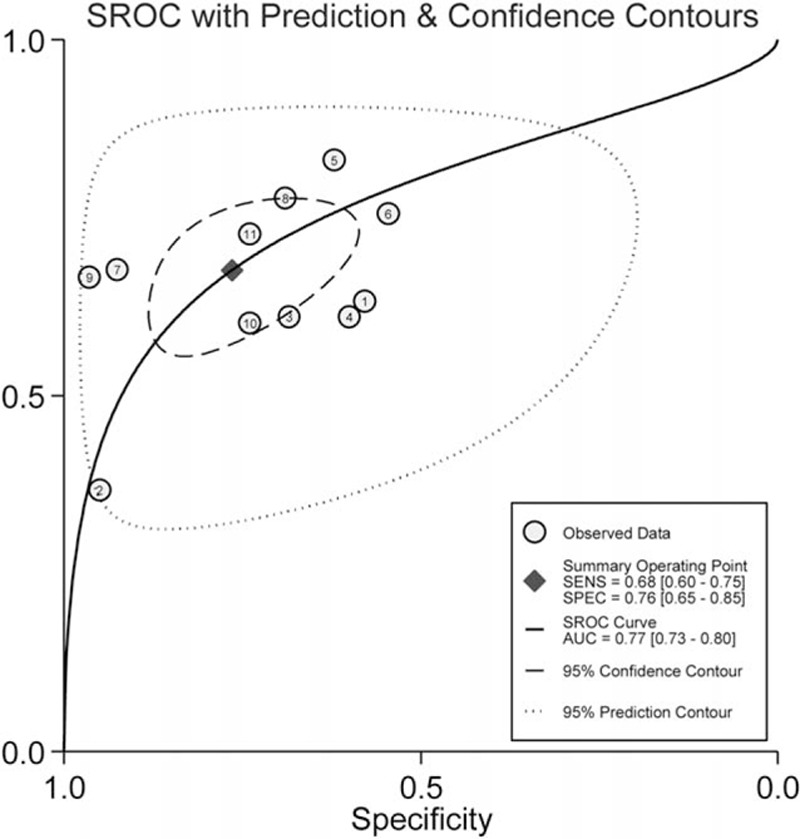
Summary receiver operating characteristic curve for miR-126 in the diagnosis of cancer.

**Figure 4:**
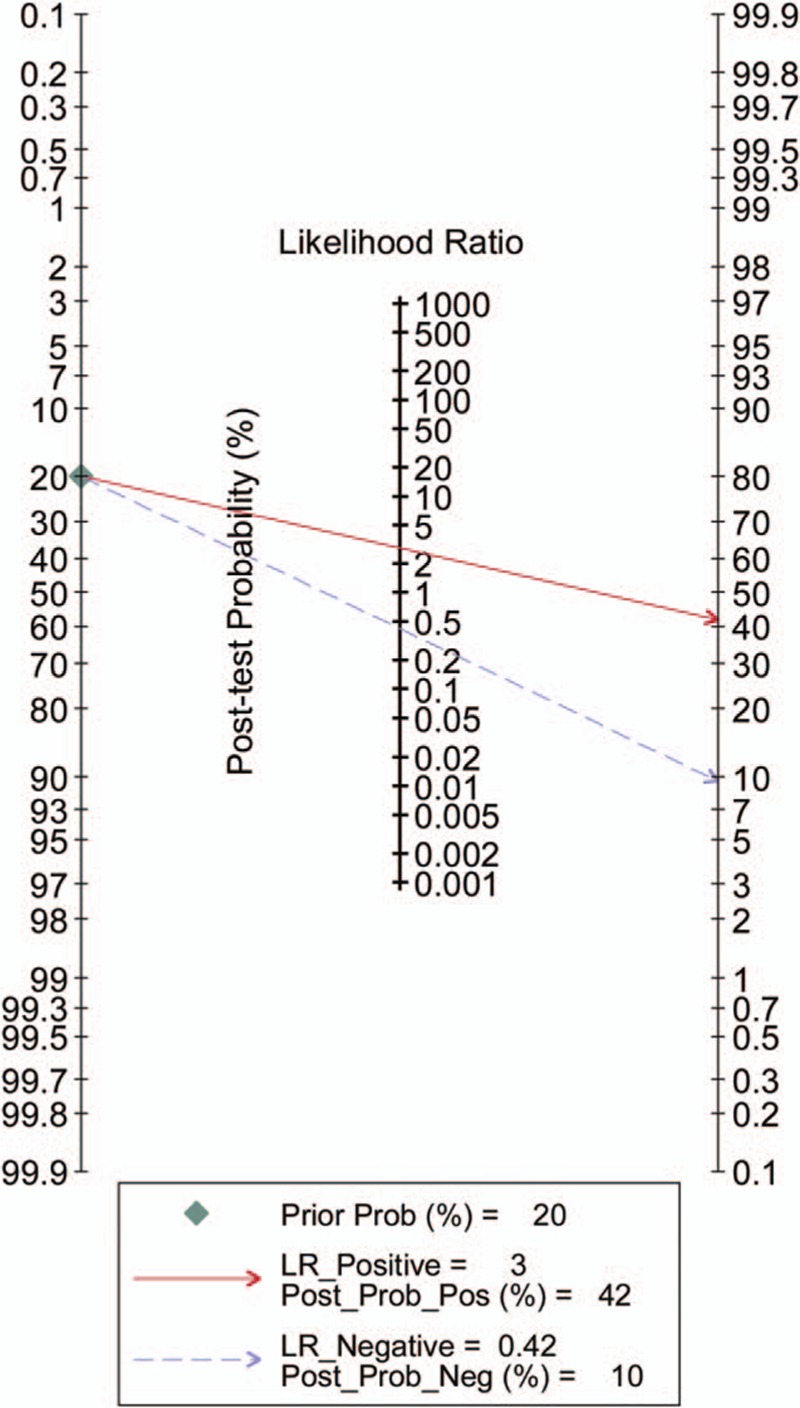
Fagan's nomogram in assessment of the test probabilities after miR-126 assay.

Heterogeneity in sensitivity and specificity were observed among the 7 studies (*I*^2^ = 80.33% and *I*^2^ = 70.12%), which indicated significant heterogeneity (Fig. 2A). Therefore, the random effects model was selected in this study. For miR-126, the sensitivity, specificity, PLR, NLR, diagnostic score, and DOR of 7 included studies were performed by forest plots. A pooled sensitivity and specificity of miR-126 were 68% (95% CI: 60–75%) and 76% (65–85%) in the diagnosis of cancer patients, respectively (Fig. 2A). Its PLR and NLR in diagnosis cancer were 2.87 (95% CI: 1.96–4.21) and 0.42 (95% CI: 0.35–0.52) separately (Fig. 2B). The diagnostic score was 1.92 (95% CI: 1.43–2.40), and the DOR is 6.79(95% CI: 4.18–11.02) (Fig. S1). The SROC curve for the included studies was shown in Fig. 3. The AUC was 0.77 (95%CI: 0.73–0.80), indicating a moderate diagnostic accuracy of miR-126 for cancer diagnosis. Furthermore, the post-test probability was calculated, and miR-126 harbored a pretest probability of 20% to have cancer. A positive result would improve post-test probability having cancer to 42%, whereas a negative result would drop the post-test probability to 10% (Fig. 4).

### 3.4 Threshold effect and heterogeneity

It is known that the heterogeneity between the studies can influence diagnosis accuracy. The threshold effect and the nonthreshold effect are 2 main sources of heterogeneity. The threshold effect is partly caused by differences of sensitivity and specificity and spearman correlation coefficient of sensitivity and specificity is often used to evaluate the threshold effect.^[31]^ In our meta-analysis, the spearman correlation coefficient of sensitivity and 1-specificity was 0.310 and *P* value was 0.354 (*P* > 0.05), identifying that there is no heterogeneity from the threshold effect. In addition, the nonthreshold effect is assessed by *I*^2^. In this study, the value of *I*^2^ >50% suggested that the heterogeneity by the nonthreshold effect is also existed among these studies.^[32]^ Then, we performed the meta-regression based on the variables including publication year, country and ethnicity, sample type, cancer type, miR-126 change tendency, sample number, and study quality, to explain this heterogeneity. Meta-regression analysis indicated that sample type, cancer type, sample number, and study quality were the sources of heterogeneity for sensitivity (Table 2). The circulating miR-126 expression change in cancer was inconsistent. Six studies from 3 papers reported that miR-126 was upregulated in cancer patients and for these 6 studies. Pooled sensitivity, specificity, PLR, NLR, DOR, and AUC were 60% (95% CI: 51%–68%), 74% (95% CI: 63%–83%), 2.3 (95% CI: 1.7–3.1), 0.54 (95% CI: 0.46–0.65), 4 (95% CI: 3–6), and 0.71(95% CI: 0.66–0.74), respectively. In contrast, miR-126 was decreased in cancer patients compared to controls in the other 5 studies and pooled sensitivity, specificity, PLR, NLR, DOR, and AUC were 76% (95% CI: 68%–82%), 80% (95% CI: 59%–92%), 3.8 (95% CI: 1.8–8.3), 0.30 (95% CI: 0.25–0.37), 13 (95% CI: 5–29), and 0.81(95% CI: 0.77–0.84), which showed higher diagnosis accuracy for miR-126.

**Table 2:**
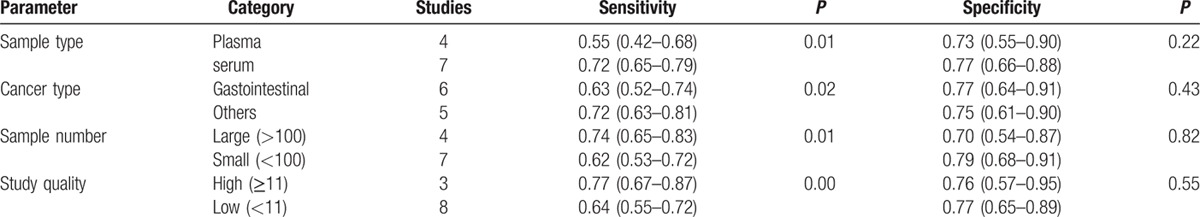
Meta-regression analysis of different parameters regarding the heterogenicity.

### 3.5 Publication bias

**Figure 5:**
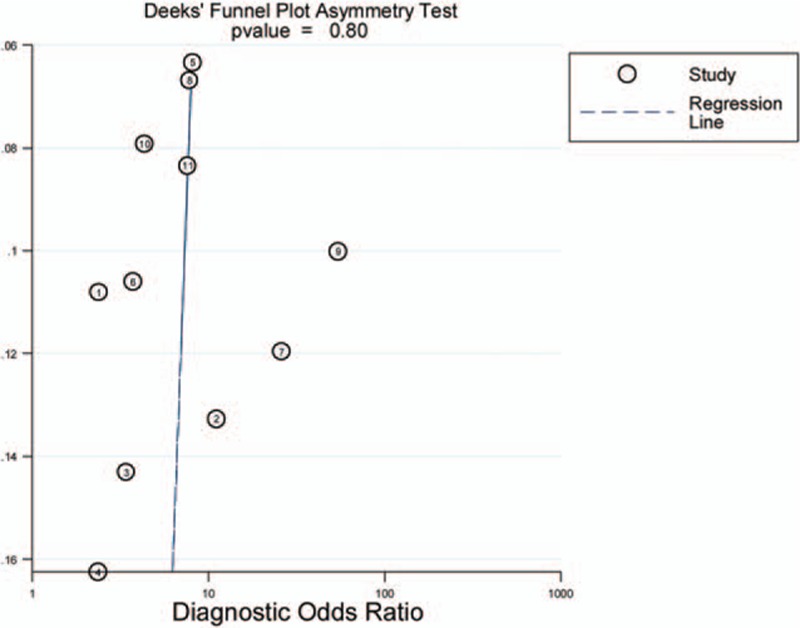
Deeks’ funnel plots asymmetry test with regression line to explore publication bias.

To assess publication bias in this study, the included studies were evaluated using Deeks’ test. As shown in Fig. 5, Deeks’ funnel plot was symmetric, and the *P* value was 0.80. Thus, there was no publication bias in this meta-analysis.

## 4 Discussion

Cancer detection methods such as cytological analysis of sputum, computed tomography, and endoscopic ultrasound-guided fine needle aspiration had a lot of limitations including low diagnostic accuracy, late diagnosis, and invasion to human body. Thus, proper and noninvasive tumor biomarkers are essential to early cancer detection and diagnosis. Cancer noninvasive biomarkers, such as CEA and CA19–9, are widely used in clinics. However, these markers lack sensitivity and specificity for cancer screening.^[33]^

It is reported that cancer is attributed to the intrinsic or exogenous genetic alterations of cells and miRNAs have been reported to be involved in the development of tumor as a regulator in gene expression.^[34]^ In addition, recent studies have confirmed that miRNAs were released from broken cells and entered into the circulation system including blood and other body fluid. The discovery of miRNAs in blood has shed new light on early diagnosis of cancer. Furthermore, plasma miRNAs were proved to be resistant to plasma RNase activity, indicating that miRNAs remain intact in plasma and are stable for detection.^[35]^ At present, many miRNAs have been reported to regulate cancer.^[36]^ Among them, miR-126 is reported to be a potential biomarker in cancer screening and has attracted more and more attention. However, due to limited case number, the results are still inconsistent. To our knowledge, we first evaluated if miR-126 could be used as a novel biomarker in cancer diagnosis.

In the present study, we found that circulating miR-126 could discriminate cancer from controls and yielded an AUC of 0.77 (95%CI: 0.73–0.80) with a sensitivity of 68% (95% CI: 60–75%) and a specificity of 76% (65–85%). An AUC of 0.93 to 1 is considered to be of very good diagnosis effect and 0.75 to 0.92 is good. This result of 0.77 suggests that miR-126 is a good potential noninvasive biomarker for cancer. The DOR is a single indicator of test accuracy that combines sensitivity and specificity. We identified that the pooled DOR was 6.79 (95% CI: 4.18–11.02), indicating that the overall accuracy of the miR-126 test for detecting cancer was relatively high. The PLR and NLR are more clinically meaningful for measures of diagnostic accuracy. The pooled PLR and NLR were 2.87 (95% CI: 1.96–4.21) and 0.42 (95% CI: 0.35–0.52) respectively. The PLR value of 2.87 suggests that patients with cancer have an approximately 2.87-fold higher chance of being miRNA-126 differently expressed compared to control patients without cancer. The NLR value of 0.42 indicates that the probability of having cancer is 42% when the miR-126 is abnormal. The miR-126 was reported upregulated in some studies; however, some literatures reported that miR-126 was decreased in some cancer patients. According to our results, downregulated miR-216 had higher diagnosis accuracy.

Heterogeneity is a potential problem that we must consider in interpretation of the results. Primary causes of heterogeneity in test accuracy studies are the threshold effect, nonthreshold effect, and publication bias. The Spearman correlation coefficient was used to analyze the threshold effect. The Spearman correlation coefficient of sensitivity and 1-specificity is 0.310 (*P* = 0.354), which indicates that there is no heterogeneity from threshold effects. After meta-regression analysis, we considered that sample type, cancer type, sample number, as well as study quality might be the possible sources of heterogeneity in the study. Studies with larger sample number, with higher study quality, and detected in serum showed higher diagnosis accuracy for miR-216, which was inspiring. In our meta-analysis, the *P* value of Deeks’ funnel plot was 0.80, so there is no risk that publication bias might adversely affect the reliability of the result. These results indicate that more studies with larger sample number, higher study quality, and consistent procedure standard are needed.

There also seemed to be some limitations in the meta-analysis. First, the study size obtained in this meta-analysis is relatively small. Therefore, further validations of miR-126 in large cohort and independent studies are needed. Second, although we have tried our best to cover all the involved literatures by a comprehensive method, we may still miss some of them during the screen process. Third, only articles written in English were included in this meta-analysis, and articles written in other languages, unpublished data and ongoing studies were not included, which may contribute to publication bias in our meta-analysis. Finally, failure to get the original data from the studies limited our meta-analysis on reliability of miR-126 diagnosis effect.

In conclusion, our meta-analysis identified that miR-126 has strong potential to be a novel noninvasive biomarker in cancer detection. Larger scale prospective studies also should be done to further validate its diagnostic effect. In future, miR-126 might be used as a cancer screening tool in clinic.

## Supplementary Material

Supplemental Digital Content
